# Commentary on Sources of Attention-Deficit/Hyperactivity Overdiagnosis Among U.S. Adults

**DOI:** 10.3390/healthcare13182367

**Published:** 2025-09-20

**Authors:** Samuel R. Weber

**Affiliations:** 1 Intermountain Health, Logan, UT 84341, USA; samuelrobertweber@gmail.com; Tel.: +1-435-716-1280; 2 Spencer Fox Eccles School of Medicine, University of Utah, Salt Lake City, UT 84113, USA

**Keywords:** ADHD, diagnosis, mental health

## Abstract

Diagnosis of adult attention-deficit/hyperactivity disorder (ADHD) has risen precipitously in recent years in the United States. This has been accompanied by a corresponding increase in rates of stimulant medication prescriptions, resulting in prescription drug shortages. These data raise concern that adult ADHD may be overdiagnosed. This article examines factors that can contribute to adult ADHD overdiagnosis. Sources of overdiagnosis include lack of adherence to DSM-5-TR diagnostic criteria, poor diagnostic practices, malingering, electronic distractions, cultural shifts in how the term “ADHD” has been used, and other health conditions that impair attention. More rigorous diagnostic practices are necessary to ensure appropriate diagnoses and treatments are offered. Adopting such practices will help optimize patient outcomes. Such practices include ruling out other conditions that impair attention, taking a careful developmental history, gathering information from collateral sources, and evaluating the patient for functional impairments.

## 1. Introduction

Attention-deficit/hyperactivity disorder (ADHD) is a condition that, if unrecognized and untreated, is associated with increased risk of substance abuse and worse functional outcomes in adulthood [1]. Additionally, females are less likely to be diagnosed with ADHD than their male counterparts due to their predominantly inattentive presentation. Such inattentive presentations may be overlooked when compared with the socially disruptive hyperactive/impulsive symptoms more commonly seen in males [2]. However, recent years have seen a trend of increasing diagnosis of ADHD among male and female adults in the United States. Diagnosis of ADHD among US adults doubled between 2007 and 2016 [3]. The United States outpaces the rest of the world in ADHD diagnoses, with an estimated 6% of US adults having received the diagnosis (with half of those being diagnosed in adulthood) in contrast to the worldwide prevalence of 2–5% [4]. Beyond those who have received the diagnosis, many Americans either suspect or self-diagnose ADHD in adulthood. A national survey of adults in 2024 found that 25% of adults suspect they have ADHD [5]. As rates of adult ADHD diagnosis have increased, so too have rates of adults receiving prescriptions for ADHD medications. Stimulant prescriptions written to young adults increased by a factor of 10 between 1994 and 2009 [6]. Prescriptions for psychostimulants increased by 30% from 2018 to 2022 (notably spanning the 2020 COVID-19 pandemic), whereas other mental health prescriptions including antidepressants, benzodiazepines, and buprenorphine remained stable [7]. The rising use of prescription stimulants led to national drug shortages, prompting the FDA and DEA to issue a joint letter in 2023 encouraging clinicians to be careful with their diagnostic practices surrounding adult ADHD [8]. These data raise the concern that adult ADHD may be overdiagnosed in the United States [9,10,11,12].

Why are ADHD diagnosis rates rising so precipitously? This article aims to review a variety of potential factors contributing to ADHD overdiagnosis in the United States. Sources of overdiagnosis can include lack of adherence to DSM-5-TR diagnostic criteria, poor diagnostic practices, malingering, electronic distractions, cultural shifts in how the term “ADHD” has been used, and other health conditions that impair attention.

## 2. DSM-5-TR Criteria

As with all mental health disorders, ADHD is defined by criteria contained within the Diagnostic and Statistical Manual for Mental Disorders, Fifth Edition, Text Revision (DSM-5-TR) [13]. The DSM-5-TR criteria for ADHD are outlined in Table 1.

Importantly, ADHD is defined as a neurodevelopmental disorder, meaning it has “onset in the developmental period… typically manifest early in development, often before the child enters school” [11] (p. 35). Adjustments to DSM criteria in recent years have likely contributed to rising rates of adult ADHD diagnosis. Prior editions of the DSM specified that symptoms must be present prior to age 7 to meet criteria for ADHD. DSM-5 changed this age-based cutoff to 12, thereby increasing the number of potential patients who qualify for the diagnosis [13]. Regardless of the age used, if this age-based requirement is ignored it can easily lead to overdiagnosis of ADHD in adults. DSM-5 also reduced the number of required symptoms for inattention or hyperactivity/impulsivity in adults from six (still the standard in children) to five, a lowered threshold that also increases the likelihood of patients meeting criteria for ADHD [13]. Many patients present for ADHD evaluations and lead with a complaint of “hyperfocus”, or episodes of highly focused attention [14]. Although patients with ADHD may experience deep, prolonged focus on a specific task or activity, difficulty shifting attention, and a lack of awareness of their surroundings, this is not a DSM-5-TR symptom of ADHD and therefore should not be used in establishing the diagnosis [13].

While the DSM provides a conceptual framework to understand mental disorders on a syndromal level, ADHD (like virtually all psychiatric disorders) is not a condition with currently identified biomarkers to enhance diagnostic accuracy. This means that its symptoms are highly subjective. As such, attempting to retrospectively diagnose a developmental condition in adulthood is frequently biased by the patient’s current level of functioning [15]. This means that if a patient is currently feeling distracted, they are more prone to recall events from their history in which they similarly felt distracted. This can lead to inappropriately medicalizing normal variants in the population [10]. Nonimpairing cognitive fluctuations can lead to false positive cases of late-onset ADHD, thereby contributing to overdiagnosis [16].

While DSM-5-TR criteria for ADHD require at least 5 inattentive and/or 5 hyperactive/impulsive symptoms, the presence of symptoms alone is not sufficient to establish the diagnosis. It is also necessary that these symptoms produce significant impairment in the patient’s functioning [17,18]. These functional impairments can include academic performance, work performance, school participation, and family functioning. It is also important to establish whether these functional impairments are present in multiple settings (e.g., school and home) [13].

## 3. Poor Diagnostic Practices

Unfortunately, there are no current professional guidelines for the assessment of adult ADHD in the United States [18]. This contributes to a milieu in which diagnostic approaches can be highly variable between clinicians [19]. Often self-reported symptom checklists such as the Adult ADHD Self-Report Scale (ASRS) are relied upon to assist in diagnosis [20]. Unfortunately, such self-reported symptoms are less reliable than reports from informants [21,22]. Clinicians, lacking established professional guidelines and who may be insufficiently trained, pressured to diagnose reimbursable conditions, and short on time, may inappropriately utilize screening questionnaires such as the ASRS as diagnostic tools. However, screening tools are high in sensitivity but low in specificity, which can lead to overdiagnosis [23]. Screeners do not establish a diagnosis, but prompt the clinician to investigate further [24]. One study showed that using the ASRS in the general population will result in 7–10 times over-identification of ADHD, and that about 90% of people who screened positive for ADHD using the ASRS were unlikely to have the condition [25].

Collateral information from third parties is more reliable than self-report in establishing the presence of ADHD [17,18]. Such sources can help distinguish ADHD from other conditions. Parents are the ideal informants, given their view of the patient’s functioning during childhood. Other potential sources of collateral include significant others, behavioral questionnaires completed by parents for current and childhood symptoms, school records, and intellectual and achievement testing. There is evidence of a familial/heritability component to ADHD, which may tempt clinicians to rely on family history in making an ADHD diagnosis [26]. However, clinical overreliance on family history in establishing a new diagnosis could provide a misleading diagnostic impression. It is often not clear what first-degree relatives have suffered from due to the absence of a formal diagnosis, patient unawareness of the diagnosis, or potential misdiagnosis of family members [27].

## 4. Malingering

Some adults may consciously malinger or feign symptoms of ADHD. This is especially common in university settings where students may be seeking accommodations [28]. One study showed that 25–48% of college students who referred themselves for an ADHD evaluation exaggerated their symptoms [29]. Another study showed that nearly a third of adults failed a credibility test assessing their performance in ADHD evaluations [30]. Those who do malinger ADHD symptoms do not have difficulty identifying and exaggerating symptoms that correspond with the DSM-5-TR criteria [31].

## 5. Electronic Distractions

Smartphones and social media likely play a role in impairing concentration. Johann Hari wrote in his book *Stolen Focus* that electronic distractions lead to increased attentional switching, less time spent in mental flow states, a reduction in sustained reading, and less time spent in beneficial mind-wandering, all of which contribute to difficulty sustaining attention [32,33,34]. A recent study blocked all mobile internet access from participants’ smartphones. After two weeks of smartphone internet abstinence, over half of participants demonstrated improved performance on objective measures of their ability to sustain attention. This change in attention was roughly the same magnitude as ten years of age-related decline [35].

Even for patients who are not consciously malingering ADHD symptoms, the way that patients perceive themselves is highly influenced by cultural discourse around ADHD. Social media is, for better or worse, a significant source of information for most patients. Studies have shown that discussions of ADHD on social media outlets are often misleading or unhelpful. One study found that among 100 TikTok videos about ADHD, over half of them were misleading, and only 21% were useful [36]. Another study found that among the top 100 TikTok videos with the hashtag “#ADHD”, fewer than half of the claims made about ADHD symptoms aligned with the DSM-5-TR diagnostic criteria. The study also showed that young adults who viewed TikTok videos about ADHD were likely to think ADHD is more common and more disabling compared to those who did not watch the videos [37]. When inaccurate social media discourse prompts patients to perceive themselves as ill and seek evaluation based on incorrect information, it can lead to overdiagnosis.

## 6. Other Health Conditions

While ADHD may be the “poster child” for impairments in attention, it is not the only condition that can impair a person’s concentration [17,18]. Other psychiatric disorders can manifest with inattention. Such conditions include anxiety disorders (including generalized anxiety disorder), chronic stress, posttraumatic stress disorder, recent trauma, major depressive disorder, bipolar disorder, personality disorders, learning disorders, and age-related cognitive decline [17,38,39]. Comorbidity of ADHD with other psychiatric disorders can complicate diagnosis and treatment [18]. Likewise, sleep disorders can negatively affect attention. Sleep deprivation, sleep apnea, restless leg syndrome, and delayed sleep phase-onset disorder all can manifest with inattentive symptoms [38,39]. There are also many substances that can impair concentration and focus, including marijuana/cannabis, cocaine, MDMA/ecstasy, caffeine, anticholinergic drugs, alcohol, and opioids [17,38]. Medical conditions that commonly affect attention include seizures, Lyme disease, HIV, delirium, hypothyroidism, “chemo brain”, obesity, poor nutrition, heart disease, hypertension, concussions, and inflammation [17,38]. There are also environmental considerations in considering a patient’s inattention, such as overdemanding environments, pollution, chemical exposures, and the aforementioned excessive use of digital media [17,39]. Normal cognitive fluctuations must also be considered [17]. Studies have shown that non-ADHD conditions such as these may account for 93–95% of cases of adult-onset inattention [39]. A thorough evaluation for adult ADHD must screen for and consider the presence of these aforementioned conditions in order to clearly establish the underlying cause of a patient’s subjective experience of inattention.

## 7. Discussion

With rates of adult ADHD rising in the United States, it is incumbent upon clinicians to be careful in their diagnostic approach. Overdiagnosing adult ADHD can lead to inappropriate or unnecessary treatments and the prescription of controlled stimulants to individuals for whom they are not indicated [40]. When evaluating an adult for potential ADHD, adherence to DSM-5-TR criteria rather than unreliable approaches such as family history or reports of hyperfocus is key. If screening questionnaires such as the ASRS are used, clinicians must keep in mind that screeners are a starting point for a more thorough diagnostic interview and do not provide the diagnosis on their own. Clinical awareness of how social media discourse around the term “ADHD” may influence a patient’s perceptions is beneficial. Clinicians need to be aware of and rule out the possibility of malingering, electronic distractions, and other health conditions, including psychiatric disorders, substance use, sleep disorders, medical conditions, and environmental factors that impair attention. If other conditions affecting attention have been identified, referrals or treatments targeting those conditions can be offered. Once other disorders have been ruled out that may present similarly to ADHD, clinicians should take a careful developmental history and ensure the presence of symptoms prior to the age of 12 which have persisted into adulthood [15,28]. Clinicians should obtain reports from collateral sources of information such as parents, school records, or significant others, which have been shown to be more reliable than patient self-report [15,21,28]. Lastly, clinicians need to assess patients for functional impairment. This means not only that a patient has a predefined number of DSM-defined symptoms, but that these symptoms have resulted in actual impairment in the patient’s academic, work, or social performance [17]. While many factors could lead to overdiagnosing ADHD in adults, recognizing these issues and actively working toward a more rigorous assessment process can improve diagnostic accuracy and patient outcomes.

## Figures and Tables

**Table 1:** Summary of DSM-5-TR diagnostic criteria for ADHD [13] (pp. 68–69).

**A.** A persistent pattern of inattention and/or hyperactivity-impulsivity that interferes with functioning or development, characterized by (1) and/or (2):	
**1. Inattention:** 6+ symptoms persisting 6+ months, inconsistent with developmental level, negative impact directly on social and academic/occupational activities (Note: For those age 17+, at least 5 symptoms are required)	**a.** Careless mistakes
	**b.** Difficulty sustaining attention
	**c.** Does not listen when spoken to directly
	**d.** Does not follow through on instructions
	**e.** Difficulty organizing tasks
	**f.** Avoids tasks with sustained mental effort
	**g.** Loses things
	**h.** Easily distracted
	**i.** Forgetful
**2. Hyperactivity and impulsivity:** 6+ symptoms persisting 6+ months, inconsistent with developmental level, negative impacts directly on social and academic/occupational activities (Note: For those age 17+, at least 5 symptoms are required)	**a.** Fidgets
	**b.** Leaves seat
	**c.** Inappropriate running or restlessness
	**d.** Unable to engage in leisure quietly
	**e.** Uncomfortable being still
	**f.** Talks excessively
	**g.** Completes others’ sentences
	**h.** Difficulty waiting turn
	**i.** Interrupts others
**B.** Several inattentive or hyperactive-impulsive symptoms present prior to age 12	
**C.** Several inattentive or hyperactive-impulsive symptoms present in 2+ settings	
**D.** Clear evidence that symptoms interfere with, or reduce the quality of, social, academic, or occupational functioning	
**E.** The symptoms do not occur exclusively during a psychotic disorder and are not better explained by another mental disorder	

## Data Availability

No new data were created or analyzed in this study. Data sharing is not applicable to this article.

## Abbreviations

The following abbreviations are used in this manuscript:

ADHD	Attention-deficit/hyperactivity disorder
ASRS	Adult ADHD Self-Report Scale
DSM-5-TR	Diagnostic and Statistical Manual for Mental Disorders, Fifth Edition, Text Revision
US	United States
